# Calls reveal population structure of blue whales across the southeast Indian Ocean and the southwest Pacific Ocean

**DOI:** 10.1093/jmammal/gyv126

**Published:** 2015-08-18

**Authors:** Naysa E. Balcazar, Joy S. Tripovich, Holger Klinck, Sharon L. Nieukirk, David K. Mellinger, Robert P. Dziak, Tracey L. Rogers

**Affiliations:** Evolution & Ecology Research Centre, School of Biological, Earth and Environmental Sciences, The University of New South Wales, Biological Sciences Building (D26), Randwick, Sydney, NSW 2052, Australia (NEB, JST, TLR); Cooperative Institute for Marine Resources Studies, Oregon State University Hatfield Marine Science Centre, 2030 SE Marine Science Drive, Newport, OR 97365, USA (HK, SLN, DKM); Bioacoustics Research Program, Cornell Lab of Ornithology, Cornell University, 159 Sapsucker Woods Road, Ithaca, NY 14850, USA (HK); Pacific Marine Environmental Laboratory, National Oceanic and Atmospheric Administration, Hatfield Marine Science Centre, 2030 SE Marine Science Drive, Newport, OR 97365, USA (RPD)

**Keywords:** Australia, bioacoustics, Lau Basin, marine mammal, New Zealand, passive acoustic monitoring, pygmy blue whale, vocalization

## Abstract

For effective species management, understanding population structure and distribution is critical. However, quantifying population structure is not always straightforward. Within the Southern Hemisphere, the blue whale (*Balaenoptera musculus*) complex is extremely diverse but difficult to study. Using automated detector methods, we identified “acoustic populations” of whales producing region-specific call types. We examined blue whale call types in passive acoustic data at sites spanning over 7,370 km across the southeast Indian Ocean and southwest Pacific Ocean (SWPO) from 2009 to 2012. In the absence of genetic resolution, these acoustic populations offer unique information about the blue whale population complex. We found that the Australian continent acts as a geographic boundary, separating Australia and New Zealand blue whale acoustic populations at the junction of the Indian and Pacific Ocean basins. We located blue whales in previously undocumented locations, including the far SWPO, in the Tasman Sea off the east coast of Australia, and along the Lau Basin near Tonga. Our understanding of population dynamics across this broad scale has significant implications to recovery and conservation management for this endangered species, at a regional and global scale.

Understanding species’ population structure is necessary for wildlife management and has great conservation implications. This is particularly important for endangered species, for which an understanding of species occurrence, range, and distribution facilitates the implementation of specific conservation objectives. Population structure can be defined as the distribution of individuals (i.e., the geographic area where animals are found and the gene flow connection between areas—Slatkin 1987; Pritchard et al. 2000). However, quantifying population structure is not always straightforward. Some species are difficult to access, such as those found in remote areas, those that are highly mobile, and those with low numbers. Under these circumstances, collecting genetic material proves challenging.

To address these challenges, differences in animal vocalizations can be used to reveal population structure (Mellinger and Barlow 2003). These “acoustic populations” use calls that are unique to their geographical distribution. Geographical variations in calls have been identified in damselfish (Mann and Lobel 1998), American pika (Conner 1982), sparrows (Baptista 1975), macaques (Fischer et al. 1998), frogs and toads (Gerhardt 1994), crickets (Ryan and Wilczynski 1991), bats (Barclay et al. 1999), Weddell seals (Thomas and Stirling 1983), leopard seals (Thomas and Golladay 1995), sperm whales (Weilgart and Whitehead 1997), killer whales (Ford and Fisher 1982), humpback whales (Winn et al. 1981; Noad et al. 2000; Cerchio et al. 2001), fin whales (Thompson et al. 1992), Bryde’s whales (Oleson et al. 2003), and blue whales (McDonald et al. 2006).

Passive acoustic monitoring (PAM) is a cost-effective method of collecting animal vocalizations over large spatial and temporal scales (Mellinger et al. 2007; Laiolo 2010). It is not limited by direct field access to animals and is not dependent on daylight. Despite PAM’s limitations (e.g., it can assess animals only when they are vocalizing), this method can provide information on species occurrence and can infer distribution and movement patterns. The time and place of calls can reveal information on migration routes and aggregation areas. This information is useful in the marine environment, particularly for migratory and endangered species, for which direct access to animals may be limited.

The blue whale (*Balaenoptera musculus*) is the largest animal alive, yet we know relatively little about its activity in the Southern Hemisphere. Their off-shore distribution and low population density make field studies and direct observation difficult. Blue whales are classified as “Endangered” under the International Union for Conservation of Nature Red List of Threatened Species (Reilly et al. 2008). Information on their abundance, occurrence, range, and distribution remains unclear (Branch et al. 2007).

Blue whales have among the loudest of animal calls at approximately 189 dB (re 1 µPa at 1 m—Sirovic et al. 2007). They produce these loud calls at low frequencies (under 100 Hz), which maximizes their detection range (Stafford et al. 1998; Sirovic et al. 2007; Stafford et al. 2007; Samaran et al. 2010a). The calls of the blue whale are stylized and repetitive, which is ideal for species and population recognition (McDonald et al. 2006) and PAM. Acoustics has played a vital role in identifying the population structure of this species. At the moment, there are 10 blue whale call types (acoustic populations) in the literature, 9 of which have been described by McDonald et al. (2006) and 1 by Frank and Ferris (2011). This is likely an underestimate as other call types and populations are being discovered (Mellinger and Barlow 2003). Seven of these call types are found in the Southern Hemisphere. In the southern Indian Ocean, 4 call types have been found: the Antarctic (Stafford et al. 2004), Sri Lankan (Stafford et al. 2011), Madagascan (Ljungblad et al. 1998), and Australian (McCauley et al. 2000) blue whale (AUSB) call types. In the southern Pacific Ocean, 3 calls types have been found: the Solomon (Frank et al. 2011), New Zealand (Kibblewhite et al. 1967; McDonald 2006), and Chilean (Cummings and Thompson 1971; Buchan et al. 2010) blue whale call types.

In the southern Indian Ocean, most blue whales are believed to move from high-latitude summer feeding grounds to low-latitude wintering grounds. Confirmed feeding sites include the western (Rennie et al. 2009) and southern coasts of Australia (Gill 2002; Gill et al. 2011) and Antarctica (Sirovic et al. 2004; Sirovic et al. 2009; Sirovic and Hildebrand 2011). Based on the presence of calls in the following areas during the summer months, potential feeding sites include Diego Garcia (Stafford et al. 2011), Madagascan Basin (Samaran et al. 2013), and the Crozet Islands (Samaran et al. 2010a). Blue whale calving grounds are surmised to be at low latitudes known from seasonal winter occurrences (Corkeron and Connor 1999; Double et al. 2014).

Even less is known about the distribution and occurrence of blue whale calls in the southern Pacific Ocean. The exception is the Chilean blue whale, found in the southeast Pacific Ocean. Similar to blue whales in the southern Indian Ocean, they feed in high latitudes during summer and autumn and then move to low latitudes during winter (Hucke-Gaete et al. 2004; Buchan et al. 2014). Few studies have been carried out in the southwest Pacific Ocean (SWPO), thus, the occurrence, distribution, and movement of both the Solomon and New Zealand blue whales (NZB) remain unclear. The only known feeding site in the SWPO is off the western coast of New Zealand (Torres 2013), while low-latitude wintering grounds remain undefined.

In this study, we aim to identify blue whale occurrence in the SWPO between Australia and New Zealand; to examine the role of the Australian continent in shaping the population structure of blue whales at the junction of the southern Indian and Pacific Oceans; and to identify seasonal distribution patterns of AUSB and NZB calls across 5 sites spanning approximately 7,370 km, from the southeast Indian Ocean (SEIO) to the SWPO. An understanding of population dynamics across this broad scale has significant implications for recovery and conservation management of this endangered species.

## Materials and Methods

### Data collection

**Fig. 1.**
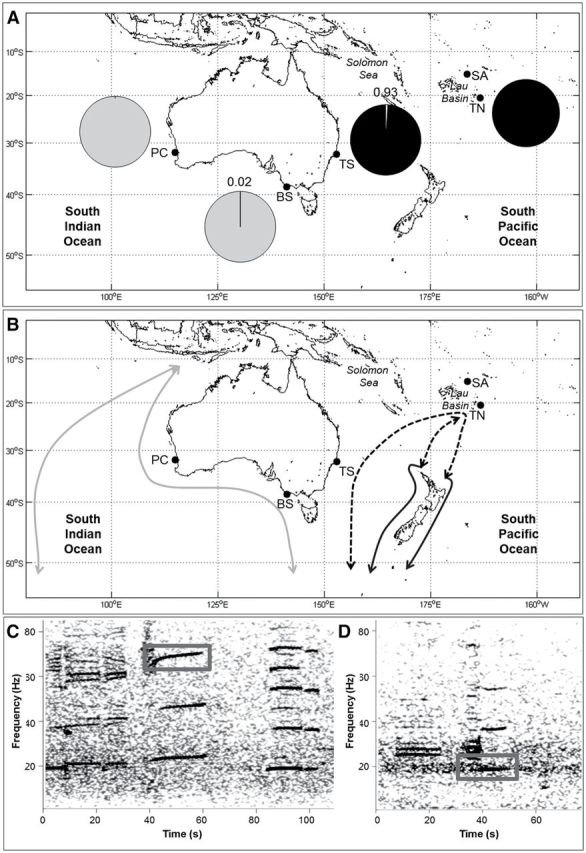
A) Proportion (%) of total number of calls found for AUSB (grey) and NZB (black) calls at each site (PC = Perth Canyon, BS = Bass Strait, TS = Tasman Sea, TN = Tonga, SA = Samoa; no calls were detected off Samoa). B) Spatial distribution and movement known for AUSB (grey), NZB (black), and predicted spatial distribution and movement of NZB (dashed black). C) AUSB and D) NZB calls, grey box shows the part of the call used in the detector. Spectrogram parameters: 1,024 points Fast Fourier Transform, 93.75% overlap, Hanning window.

To detect the presence of AUSB and NZB call types, passive acoustic data were collected at 5 sites spanning about 7,370 km. In the SEIO, data were collected at 2 sites approximately 2,500 km apart: Perth Canyon off western Australia and Bass Strait off southern Australia. In the SWPO , data were collected at 3 sites approximately 4,820 km apart: the Tasman Sea off eastern Australia, as well as the northern and southern positions of Lau Basin 670 km apart, the Tonga and Samoa sites (Table 1; Figs. 1a and 1b). At the Perth Canyon, Bass Strait, and Tasman Sea sites, single fixed hydrophones (from the Australian Integrated Marine Observer System) were used to record ocean sounds for 500 s of every 900 s at a sampling rate of 6,000 Hz (upper frequency limit of 2,800 Hz at −3 dB). Acoustic data were collected between January and December (2009–2012; Table 1). At the Tonga and Samoan sites, single moored autonomous hydrophones (developed by Oregon State University and NOAA/PMEL) recorded continuous ocean sounds at a sampling rate of 250 Hz off Tonga (upper frequency limit of 110 Hz at ± 3 dB) and 1,000 Hz off Samoa (upper frequency limit of 440 Hz at ± 3 dB). Acoustic data were collected near Tonga between February and September 2009 and near Samoa from January 2010 to August 2011 (Table 1).

**Table 1.** Hydrophone deployments used for a) geographical presence and b) interannual differences analysis across the southeast Indian Ocean (SEIO) and the southwest Pacific Ocean (SWPO). Days = number of days data were available. Detector rates = missed call rate, false detection rate, for AUSB and NZB.

Ocean basin	Site name	Location	Instrument depth (m)	Recording dates	Days	Detector rates AUSB (%)	Detector rates NZB (%)
(a) Geographical presence							
SEIO	Perth Canyon	−31°54′8.34″S, 115°1′36.42″E	465	February 2010– September 2010	256	7.9, 18.7	
SEIO	Bass Strait	−38°33′1.86″S, 141°15′13.92″E	168	February 2010– September 2010	236	8.7, 36.6	0, 99.9
SWPO	Tasman Sea	−32°19′21.72″S, 152°56′40.32″E	147	February 2010– September 2010	233	0, 99.5	4.6, 75.4
SWPO	Tonga	−20°25′44.64″S, 176°47′39.06″W	1,042	February 2009– September 2009	242		0.3, 80.5
SWPO	Samoa	−15°8′30.12″S, 173°44′18.72″W	1,031	February 2010– September 2010	242		0, 100

(b) Interannual differences							
SEIO	Perth Canyon	−31°54′8.34″S, 115°1′36.42″E	465	February 2009– June 2012	953	6.7^a^, 17.6^a^	
SEIO	Bass Strait	−38°33′1.86″S, 141°15′13.92″E	168	May 2009– November 2012	860	9.4^a^, 21.1^a^	0, 100
SWPO	Tasman Sea	−32°19′21.72″S, 152°56′40.32″E	147	February 2010–December 2011	506	0, 99.7^a^	5.8^a^, 87^a^
SWPO	Samoa	−15°8′30.12″S, 173°44′18.72″W	1,031	January 2010– August 2011	575		0, 100

^a^Average value for time period.

### Call detection

Long-term spectral averages, using Triton V.1.80 (Wiggins et al. 2010), a MATLAB-based software package (MATLAB 2012b), were initially used to screen for the presence of any blue whale calls. To date, no research has been conducted on blue whales in the Tasman Sea or in the regions to the north along the Lau Basin (off Tonga and Samoa). AUSB and NZB blue whale calls were the only blue whale calls detected in the SWPO long-term spectral averages. Detector templates were created in Ishmael (V.2.3.1—Mellinger 2001) for AUSB and NZB call types, based on specific frequency and time characteristics unique to each call type.

The detector for the AUSB call targeted the high-intensity 3rd harmonic of the 2nd part of the call, between 65 and 71 Hz and approximately 15 s in duration (Fig. 1c). The NZB call detector targeted the fundamental frequency, between 17 and 20 Hz in the 3rd part of the call and approximately 20 s in duration (Fig. 1d). The frequency of blue whale calls has been decreasing by about 0.14 Hz (AUSB—Gavrilov et al. 2011) to 0.16 Hz (NZB—Miller et al. 2014) per year. Thus, the bandwidth of each detector was customized to account for a decrease of approximately 0.14–0.16 Hz in frequency per year over the time of the study.

The AUSB and NZB call detectors were run across each site (Perth Canyon, Bass Strait, Tasman Sea, Tonga, and Samoa). An automated spectrogram correlation method (Mellinger and Clark 2000) was used to detect AUSB and NZB call types in Ishmael. All calls that were positively detected were checked manually by using Osprey, a Matlab program (spectrogram parameters were as follows: 1,024 points Fast Fourier Transform, 93.75% overlap, and Hanning window—Mellinger 1994). All positively detected calls were confirmed either as AUSB or NZB calls or a false detection. False detections, or calls that were not AUSB or NZB whale calls, were expressed as a percentage of the total number of automated detections. The call count was conservative and did not account for calls that may have been masked by background noise, missed during high-density calling periods when calls overlapped, or for “non-vocal” individuals that may have been in the area. Missed calls were expressed as percentages of the total number of AUSB and NZB whale calls in the data set that were missed by the automated detector. The missed call rate was calculated by comparing the number of calls picked manually to the number of calls detected automatically. Calls were checked manually and the process was repeated for 12 randomly selected days, 1 day selected for each month of the year.

### Data analysis

#### Geographic presence of AUSB and NZB call types.

Data were analyzed over an 8-month period (February to September; Table 1a) to examine the geographic distribution of AUSB and NZB call types. This time period was limited by the availability of acoustic data collected at the Tonga site. The detected AUSB and NZB calls were counted, and a proportion of each call type was calculated for each site (Perth Canyon, Bass Strait, Tasman Sea, Tonga, and Samoa). To account for different sampling methods, box plots were used to show the distribution of calls per month for each call type (AUSB and NZB). AUSB and NZB call types, detected per month, were plotted as medians with 0.25 quantiles for each site (Perth Canyon, Bass Strait, Tasman Sea, Tonga, and Samoa). A negative binomial generalized linear model was used because of the zero inflated data, since no blue whale calls were detected in some months. An analysis of variance and a post-hoc Tukey test were run on the model to examine differences in calls detected among months observed at each site. Analyses were performed using R version 3.1.0 (R Core Team 2013).

#### Interannual differences.

To identify interannual patterns of blue whale calls (AUSB and NZB), the number of calls detected at each location was compared. Interannual calls detected were compared only when more than 1 year of data were available, as for the Perth Canyon, Bass Strait, Tasman Sea, and Samoa (Table 1b). Up to 4 years of data were available for the Perth Canyon (February 2009 to June 2012) and Bass Strait (May 2009 to November 2012) sites. Up to 2 years of data were available for the Tasman Sea (February 2010 to December 2011) and Samoa (January 2010 to August 2011) sites. Only 1 year of data were available at the Tonga (2009) site, so no interannual patterns were identified. Blue whale calls (AUSB and NZB) detected per month were plotted as medians, with 0.25 quantiles for each site (Perth Canyon, Bass Strait, and Tasman Sea).

## Results

### Geographic presence of Australian and New Zealand call types

A total of 48,771 AUSB and NZB calls were detected at 4 of the 5 sites distributed across the Perth Canyon, Bass Strait, Tasman Sea, and Tonga between February and September 2009 or 2010 (Table 1a). No AUSB or NZB calls were detected at the northernmost site, Samoa. AUSB calls were predominantly found in the SEIO, at the Perth Canyon and Bass Strait sites (Fig. 1a). NZB calls were predominantly present in the SWPO, in the Tasman Sea and Tonga sites (Fig. 1a). NZB calls were much less common overall (0.7%) than AUSB calls (99.3%). The false and missed call detection rates, for the AUSB and NZB call detectors, are reported in Table 1.

#### Southeast Indian Ocean.

**Fig. 2.**
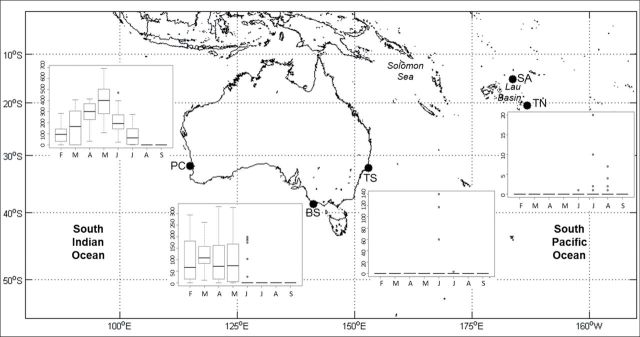
Box plots represent the median (with 0.25 and 0.75 quantile) number of calls detected per month (February to September) for AUSB at the PC = Perth Canyon and BS = Bass Strait and NZB at the TS = Tasman Sea and TN = Tonga. No AUSB or NZB whale calls were detected off SA = Samoa. Bars indicate maximum and minimum values and outliers are plotted as individual points.

In the Perth Canyon, only AUSB calls (35,635) were detected from summer (February) through winter (July) 2010. The number of calls detected varied between months, with a clear seasonal trend. Calls began to increase in February, peaked in May, and then decreased until July (Fig. 2). No calls were detected during August and September (Fig. 2). There were significantly more calls detected in April and May (Tukey’s post-hoc test, *P* < 0.001) than in any other months and significantly more calls detected in May than in April (Tukey’s post-hoc test, *P* < 0.01; Fig. 2).

In the Bass Strait, only 3 of the 12,765 calls identified were NZB calls (0.02%), while the rest were AUSB calls (Fig. 1a). NZB calls were detected across 1 day in autumn (March) 2010. AUSB calls were detected from summer (February) through winter (June) 2010, with no calls detected from July to September. The number of calls detected varied between months, as seen in the Perth Canyon, although no clear seasonal trend was evident (Fig. 2). The number of calls detected remained near constant from February to May and became sparse in June (Fig. 2). There was no significant difference between calls detected between February and May (Tukey’s post-hoc test, *P* > 0.05; Fig. 2) and significantly fewer calls detected in June than in any other month (Tukey’s post-hoc test, *P* < 0.05).

#### Southwest Pacific Ocean.

At the Tasman Sea site, only 3 of 323 calls were identified as AUSB calls (0.93%), and the rest were NZB calls (Fig. 1a). AUSB calls were detected across 1 day in autumn (May) 2010. All NZB calls were detected in winter, with virtually all calls detected in July and a few in June 2010 (Fig. 2). Off Tonga, 48 NZB calls were the only calls detected. Calls were detected only on a few occasions during the winter between June and August 2009 (Fig. 2). There was no significant difference in the number of calls detected among months (analysis of variance: *F*_7, 234_ = 1.999, *P* = 0.056) or between months (Tukey’s post-hoc test, *P* > 0.05; Fig. 2).

### Interannual differences

#### Southeast Indian Ocean.

**Fig. 3.**
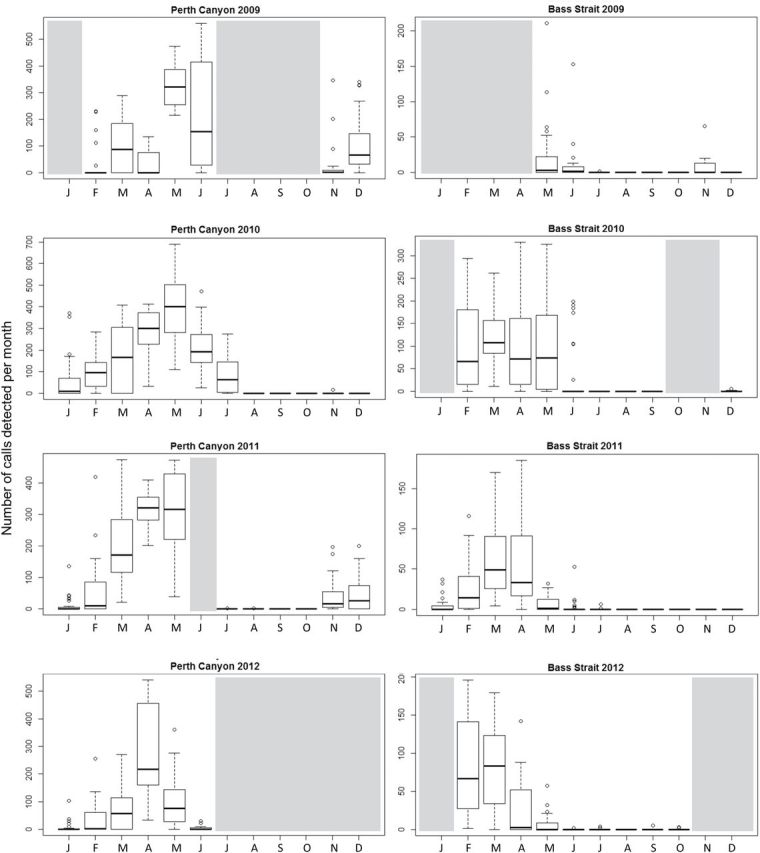
Box plots represent the median (with 0.25 and 0.75 quantile) number of AUSB calls per month (January to December) between 2009 and 2012 in the Perth Canyon and Bass Strait. Bars indicate maximum and minimum values and outliers are plotted as individual points. Grayed out months indicate when data were not available.

Across the 4-year period from 2009 to 2012 (Table 1b), AUSB calls were the only calls detected in the Perth Canyon. Although the number of calls detected in the Perth Canyon differed within and between years, there was a general seasonal trend. Calls were detected from spring (November) through winter (June, July). Calls increased from late spring to early summer, peaked in autumn, and decreased in winter (Fig. 3). In the Bass Strait, 4 years of data from 2009 to 2012 (Table 1b) showed that NZB calls were detected on a single day in autumn (March) 2010. AUSB calls varied within and between years, without a clear seasonal trend. AUSB calls were detected during summer (February) to winter (June), with a peak in the number of calls detected in autumn (March), as shown in Fig. 3.

#### Southwest Pacific Ocean.

**Fig. 4.**
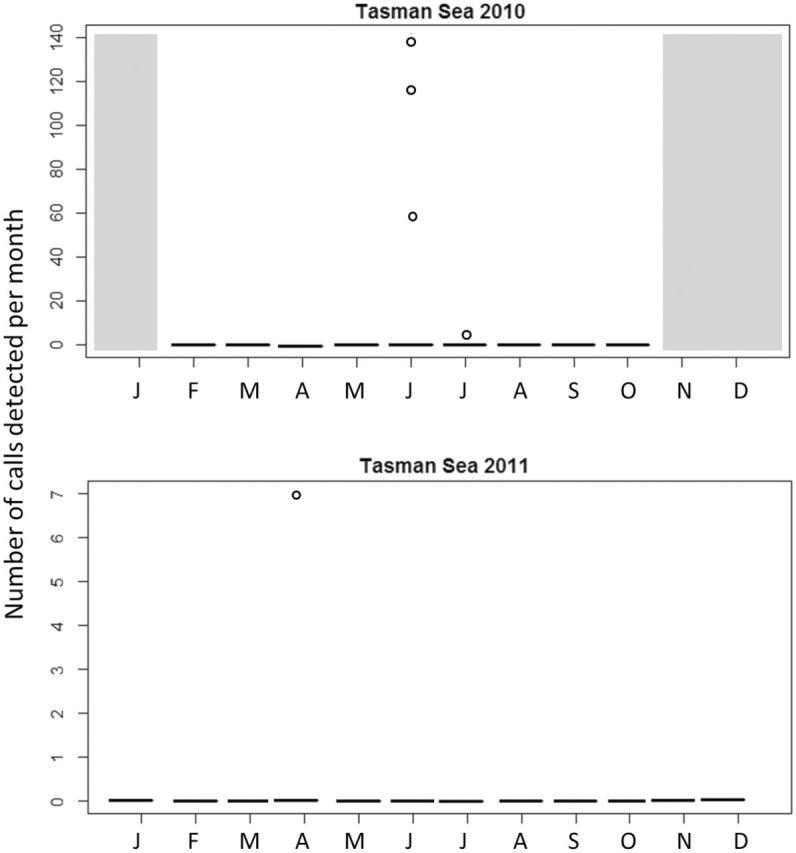
The distribution of individual NZB calls per month (January to December) between 2010 and 2011 in the Tasman Sea. Grayed out months indicate where data were not available.

In the Tasman Sea, the AUSB call was detected on a single day in autumn (May) 2010. NZB calls were detected over a few days in winter (June and July) 2010 and autumn (April) 2011 (Fig. 4). No AUSB or NZB whale calls were detected in the Samoa site in 2010 or 2011.

## Discussion

This is the first study to record blue whale calls off the Tasman Sea and Tonga. It is also the first to identify blue whale population structure across the junction of the Indian and Pacific Ocean basins. We suggest that the distribution limit for the AUSB and NZB acoustic populations is off southeastern Australia, and that the Australian continent acts as a geographic boundary segregating the AUSB and NZB call types.

This is also the first study to record NZB call types outside of New Zealand waters. NZB calls were detected in the Tasman Sea, off the Australian coastline, and north in waters around Tonga. This extends the distribution of the NZB call type from New Zealand to eastern Australia and north to Tonga (Fig. 1b). The eastern limit of this acoustic population remains unclear. NZB calls have been previously detected off the New Zealand coast (Kibblewhite et al. 1967; McDonald 2006; Miller et al. 2014) and in sub-Antarctic waters south of New Zealand (Miller et al. 2014).

NZB call types were detected in early winter (June) in the Tasman Sea and in later winter (July–August) off Tonga. Off northern New Zealand, NZB calls were previously detected on a few (4) occasions through winter (June and July), spring (September), and summer (December—McDonald 2006). Off southern New Zealand, calls were detected between summer (January) and autumn (March) during surveys conducted from January to February and in mid-March (Miller et al. 2014). This may suggest some movement of NZB from northern areas during winter to southern areas during summer. Visual surveys and stranding data have shown blue whales present along the New Zealand coast throughout the year, although no acoustic recordings have been made in this area (Torres 2013). To date, the only known feeding site in the SWPO is a coastal upwelling off the western coast of New Zealand (Torres 2013). This feeding site may explain the year-round presence of blue whales in the area. Other blue whale populations have been shown to remain year round in suggested feeding sites (Branch et al. 2007), particularly off the Costa Rica Dome (Reilly and Thayer 1990; Stafford et al. 1999), northern Indian Ocean (Anderson et al. 2012), central Indian Ocean (Stafford et al. 2011; Samaran et al. 2013), Crozet Islands (Samaran et al. 2010b), and West Antarctic (Sirovic et al. 2004).

We detected AUSB call types in the Bass Strait off southeastern Australia and in the Perth Canyon off western Australia. AUSB are known to occur off southern (Gill 2002; Gavrilov et al. 2011) and western (McCauley et al. 2000) Australia, as far west as the Crozet Islands (46°25′S, 51°40′E—Samaran et al. 2010b), north to Indonesia (Double et al. 2014), and south to sub-Antarctic waters (~54°S—Gedamke et al. 2007). Gill et al. (2011) proposed that the eastern distribution limit of the AUSB was off the Bass Strait, and this is the first study to confirm this.

In the SEIO, AUSB calls were detected in the Perth Canyon and Bass Strait sites from the summer to the beginning of winter, consistent with occurrence patterns identified from other acoustic (McCauley et al. 2000; Samaran et al. 2010a; Gavrilov et al. 2011; Stafford et al. 2011; Gavrilov and McCauley 2013; Samaran et al. 2013), visual (Gill 2002; Rennie et al. 2009; Gill et al. 2011), and tracking studies (Double et al. 2014). AUSB whales are believed to move generally from low latitude waters off Indonesia, where they spend winter and spring (Double et al. 2014), to feeding sites in coastal upwelling sites off Australia, where they spend summer and autumn (Gill 2002; Rennie et al. 2009; Gill et al. 2011). To date, only a few AUSB whales have been found (through genetic studies) in Antarctic waters (Attard et al. 2012).

Our study found that the number of AUSB and NZB calls detected was seasonal and varied between years. This pattern is common among baleen whales (Moore et al. 1998; Stafford et al. 2001; Sirovic et al. 2004; Stafford et al. 2007; Munger et al. 2008; Samaran et al. 2013). Previous studies have shown that the presence of blue whales in feeding grounds is correlated with seasonal environmental variability (e.g., differences in chlorophyll concentration and water temperature) that affect prey availability and distribution (Reilly and Thayer 1990; Fiedler et al. 1998; Rennie et al. 2009; Gill et al. 2011).

The detection of NZB calls at different latitudes suggests a possible migration route (Fig. 1b) between northern Tonga (winter breeding time, our study) and New Zealand (summer feeding area—Torres 2013; Miller et al. 2014) and between Tonga (winter breeding—our study) and the Tasman Sea (beginning of winter—our study). The Tasman Sea may be a migratory corridor for NZB, which move between low-latitude wintering grounds and high-latitude feeding grounds (Fig. 1b). NZB calls have been detected in the waters south of New Zealand at approximately 53°S (Miller et al. 2014).

Although a highly mobile species, some blue whales show site fidelity to migratory destinations (Mate et al. 1999; Galletti Vernazzani et al. 2012; Costa-Urrutia et al. 2013). This behavior is typical in other baleen whale species, like the humpback (Murray et al. 2012) and fin (Clapham et al. 1991). The distinct geographic variation in blue whale call types, even within oceans (4 blue whale call types are described within the Indian Ocean—Stafford et al. 2011; Samaran et al. 2013), suggests that southern blue whales also have site fidelity. This is not to say that all whales stay within 1 region. There is a small amount of interchange between populations, as seen in the humpback whale between the western and eastern Australian populations (Noad et al. 2000). Similarly, we detected AUSB calls over a short period (1 day) in the Tasman Sea site (typically the NZB call type site), and we detected NZB calls on 1 day in the Bass Strait site (typically the AUSB call type site). This interchange between populations (i.e., movement of a few individuals) has been shown in acoustic studies of humpback whales (Noad et al. 2000) and genetic studies of humpback (Baker et al. 1998) and southern right whales (Carroll et al. 2011). To date, no study has examined the segregation of blue whale populations at this junction.

We detected low numbers of NZB calls at the Tasman Sea site. A possible explanation for this is that the Tasman Sea is the western distribution limit of the NZB population and is not a major migratory route. This is seen in blue whale calling patterns in the Indian Ocean, where calling rates decrease as the distance from the geographic origins of the call type increases (Samaran et al. 2013). Local environmental conditions also may have influenced the probability of detection and thus resulted in low detection rates (Miller et al. 2014). Alternatively, the low number of NZB calls in the Tasman may indicate a small NZB population, such as a remnant population that has not recovered from historical whaling. Whaling records show that blue whales were caught in the Tasman Sea and in waters northwest of New Zealand; however, the original population size is unknown. Few historical accounts (sightings and strandings) were recorded, despite search efforts in the area (Branch et al. 2007). Whales may be reoccupying areas that were depleted during commercial whaling, as has been observed in some humpback (Zerbini et al. 2004), right (Groch et al. 2005), and gray whales (Bryant et al. 1984). As blue whale numbers steadily increase (Branch et al. 2004; Branch et al. 2007; Monnahan et al. 2014), an understanding of occurrence and distribution is essential, particularly in areas previously not studied.

Although we only detected AUSB and NZB calls across our study area, this does not preclude further overlap in acoustic populations. No calls were found in our northernmost site, Samoa, possibly because of its proximity to the Solomon Sea, where Frank and Ferris (2011) described the presence of the Solomon blue whale call. To clarify blue whale population structure in the greater southern Pacific Ocean, we recommend exploring the occurrence and distribution of the Solomon call type.

The spatial and temporal variation we found across and within years suggest long-term studies are necessary for clarifying distribution limits, migration routes, and seasonal patterns. Long-term studies may also help to monitor population recovery through abundance estimates over time. Ideally, a multidisciplinary approach would include genetic and morphological studies to clarify population structure and spatial–temporal boundaries.

Using PAM, we identified the acoustic population structure and distribution limits of the blue whale across the junction of the Indian and Pacific Ocean basins. We described a possible blue whale migration corridor off these areas. Understanding species population structure (i.e., occurrence and spatial–temporal distribution) is important for informed species management and conservation. This information can facilitate population-level management with specific conservation objectives. Long-term data collection may also help to monitor recovering populations and to monitor and mitigate threats or disturbance.
